# Novel Mouse Cell Lines and In Vivo Models for Human High-Grade Neuroendocrine Lung Carcinoma, Small Cell Lung Carcinoma (SCLC), and Large Cell Neuroendocrine Carcinoma (LCNEC)

**DOI:** 10.3390/ijms242015284

**Published:** 2023-10-18

**Authors:** Enrique Recuero, Sara Lázaro, Corina Lorz, Ana Belén Enguita, Ramón Garcia-Escudero, Mirentxu Santos

**Affiliations:** 1 Molecular Oncology Unit, Centro de Investigaciones Energéticas, Medioambientales y Tecnológicas (CIEMAT), 28040 Madrid, Spain; enrique.recuero@externos.ciemat.es (E.R.); saralazaro@iib.uam.es (S.L.); clorz@ciemat.es (C.L.); ramon.garcia@ciemat.es (R.G.-E.); 2 Institute of Biomedical Research Hospital “12 de Octubre” (imas12), 28041 Madrid, Spain; 3 Tumor Progression Mechanisms Program, CIBERONC, Centro de Investigación Biomédica en Red de Cáncer, 28029 Madrid, Spain; 4 Pathology Department, University Hospital “12 de Octubre”, 28041 Madrid, Spain; abenguita.hdoc@salud.madrid.org

**Keywords:** SCLC, LCNEC, cell lines, allograft, syngeneic

## Abstract

There is a clear need to expand the toolkit of adequate mouse models and cell lines available for preclinical studies of high-grade neuroendocrine lung carcinoma (small cell lung carcinoma (SCLC) and large cell neuroendocrine carcinoma (LCNEC)). SCLC and LCNEC are two highly aggressive tumor types with dismal prognoses and few therapeutic options. Currently, there is an extreme paucity of material, particularly in the case of LCNEC. Given the lack of murine cell lines and transplant models of LCNEC, the need is imperative. In this study, we generated and examined new models of LCNEC and SCLC transplantable cell lines derived from our previously developed primary mouse LCNEC and SCLC tumors. RNA-seq analysis demonstrated that our cell lines and syngeneic tumors maintained the transcriptome program from the original transgenic primary tumor and displayed strong similarities to human SCLC or LCNEC. Importantly, the SCLC transplanted cell lines showed the ability to metastasize and mimic this characteristic of the human condition. In summary, we generated mouse cell line tools that allow further basic and translational research as well as preclinical testing of new treatment strategies for SCLC and LCNEC. These tools retain important features of their human counterparts and address the lack of LCNEC disease models.

## 1. Introduction

High-grade neuroendocrine lung carcinomas are among the most malignant lung tumors. They consist of small cell lung carcinoma (SCLC) and large cell neuroendocrine carcinoma (LCNEC) and account for >15% and 2–3% of all lung tumors, respectively [1,2]. They both are neuroendocrine tumors but differ in histological characteristics. SCLC is distinguished from LCNEC by its small cell size, uniform cells, dense proliferation of small tumor cells, scant cytoplasm, higher nuclear-to-cytoplasmic ratio, inconspicuous nucleoli, and nuclear molding. LCNEC features a large cell size with organoid or trabecular patterns, rosette-like structures, abundant cytoplasm, and prominent nucleoli. It displays a greater degree of cellular pleomorphism. Their diagnosis is based on the identification of their neuroendocrine morphology and the expression of at least one of the neuroendocrine markers such as neural cell adhesion molecule (NCAM), chromogranin A (ChgA), or synaptophysin by immunohistochemical staining. Thyroid transcription factor-1 (TTF-1) is also positive in most cases [2,3]. Recently, in an attempt to better understand their nature and identify therapeutic vulnerabilities and select best treatment approaches, comprehensive molecular analyses of SCLC and LCNEC revealed differences at the genomic and transcriptomic level, leading to the identification of four molecular SCLC subtypes according to the expression of major transcription factors [4,5] and two distinct LCNEC genomic subgroups with specific transcriptional patterns named type I and type II [6]. This distinction could allow for more personalized treatments. The LCNEC group is heterogeneous, consisting of both tumors that harbor mutations found in SCLC but with an expression pattern typical of non-small cell lung carcinoma (NSCLC) and tumors with mutations found in NSCLC but with an expression pattern typical of SCLC, raising concerns about their clinical treatment as NSCLCs or SCLCs [7].

The prognosis of patients is dismal. Most SCLC or LCNEC patients are diagnosed at advanced stages, when surgery is not a therapeutic option and survival rates at 5 years are as low as 7–8% [8,9] or even less [1,10]. Although new therapeutic approaches have been developed in recent years, especially immunotherapies, these novel therapeutic strategies, if available, do not provide a good clinical outcome for many patients [1,2,11,12]. For both diseases, there is a worrisome paucity of human material from surgery; this is also due to the rarity of the tumor (LCNEC). Thus, there is a clear need to expand appropriate in vivo models that faithfully mimic the main features of the human counterparts to identify new vulnerabilities and to overcome immunotherapy resistance in SCLC and LCNEC. For LCNEC, the scarcity of preclinical models makes disease analysis and research even more challenging. 

Researchers have developed and analyzed several genetically engineered mouse models (GEMMs) of SCLC [1,4,13,14], murine cell lines, and sources of subcutaneous transplantation tumors [15,16], patient-derived (PDX) [17,18] and circulating tumor cell-derived (CDX) [19], including our own K5-QKO SCLC mouse model, in which specific ablation of the four tumor suppressors *Rb1*, *Rbl1*, *Pten*, and *Trp53* in basal epithelial airway keratin K5 expressing cells led to SCLC with a remarkable resemblance to its human counterpart [14,20,21]. Meanwhile, the situation for LCNEC is the opposite, with an evident lack of material for the study of the disease. To our knowledge, we recently developed the first and only mouse model for LCNEC [13,21] caused by the deletion of the four tumor suppressors *Rb1*, *Rbl1*, *Pten*, and *Trp53* in a wide variety of lung epithelial cells (using an adeno-CMVcre virus). Therefore, there is an urgent need to expand the availability of experimental preclinical in vitro and in vivo models for this lung cancer tumor type.

In this study, we developed novel in vitro and in vivo models of high-grade neuroendocrine carcinoma of the lung. We established, from the abovementioned spontaneous tumors, new SCLC and LCNEC cell lines that can be successfully transplanted into immunodeficient or immunocompetent mice and develop the corresponding SCLC or LCNEC tumor allografts. The derived cells and tumors maintained neuroendocrine properties and transcriptomic characteristics of the tumors from which they originated and displayed strong similarities to their human counterparts. Moreover, the SCLC syngeneic model showed the ability to develop metastases, mimicking this characteristic of the human disease. These cell lines might become robust tools for basic and translational research of high-grade neuroendocrine lung cancers as well as preclinical studies of novel therapeutic strategies. In addition, the syngeneic immunocompetent mice may be particularly suitable for testing novel immunotherapeutic and anti-metastatic approaches. 

## 2. Results

### 2.1. Generation of LCNEC and SCLC Cell Lines

**Figure 1:**
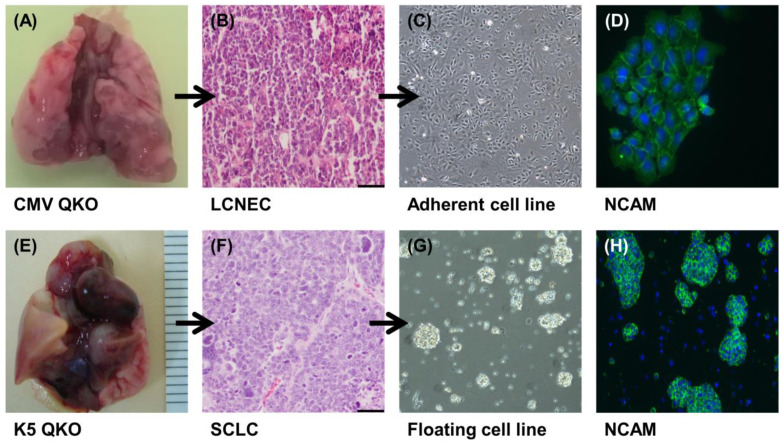
Establishment of new LCNEC and SCLC cell lines derived from CMV-QKO and K5-QKO mouse models. Gross appearance of the primary tumors in the lungs of the CMV-QKO (**A**) or K5-QKO mice (**E**). H&E staining of the primary tumors showing the characteristics of LCNEC (**B**) and SCLC (**F**), respectively. Morphology of LCNEC cells growing in a monolayer ((**C**) 4× light-field image) or SCLC cells growing in suspension ((**G**) 10× light-field image). Positive immunofluorescence staining of the neuroendocrine marker N-CAM in LCNEC (**D**) or SCLC (**H**), respectively. Scale bars = 50 µm.

The intratracheal administration of adenovirus Ad-CMVcre to adult QKO mice produced LCNEC in lungs [21]. An example of a solid tumor can be seen in Figure 1A. A representative hematoxylin and eosin staining (H&E) is shown in Figure 1B. From these tumors, we established five highly proliferative LCNEC cell lines (Figure S1A) that we named 46LCNEC, 46L2 LCNEC, 46L3 LCNEC, 47T2 LCNEC, and 47T3 LCNEC. LCNEC cells presented an adherent phenotype, growing in a monolayer with visible cytoplasm (Figure 1C,D). In parallel, SCLC tumors arose in QKO mice after the intratracheal administration of adenovirus Ad-K5cre (Figure 1E,F). Three cell lines (named 54SCLC, 57SCLC, and 61SCLC) were obtained from these tumors. SCLC cells grew rapidly (Figure S1B) as compact, suspending aggregates of very small cells, showing the same morphology as other mouse and human floating SCLC cells [22,23] (Figure 1G,H).

All mouse cell lines generated in the laboratory were authenticated by genotyping for the mutant alleles and the expression of neuroendocrine markers. Cell lines were assayed for the anticipated genetic rearrangements by PCR of genomic DNA, as previously described [21,22]. PCR primer sequences are shown in Table S1. As expected, all LCNEC and SCLC cell lines showed genetic recombination/gene deletion at the *Rb1*, *Rbl1*, *Pten*, and *Trp53* loci (Figure S2).

To better characterize these cells, we looked for different neuroendocrine marker expressions by immunofluorescence. We found cells expressing the neuroendocrine markers NCAM (Figure 1D,H), SSTR2, and CGRP (Figure S3). These observations underline the neuroendocrine properties of the cells.

### 2.2. Evaluation of In Vivo Tumorigenic Potential of LCNEC and SCLC Cell Lines 

**Figure 2:**
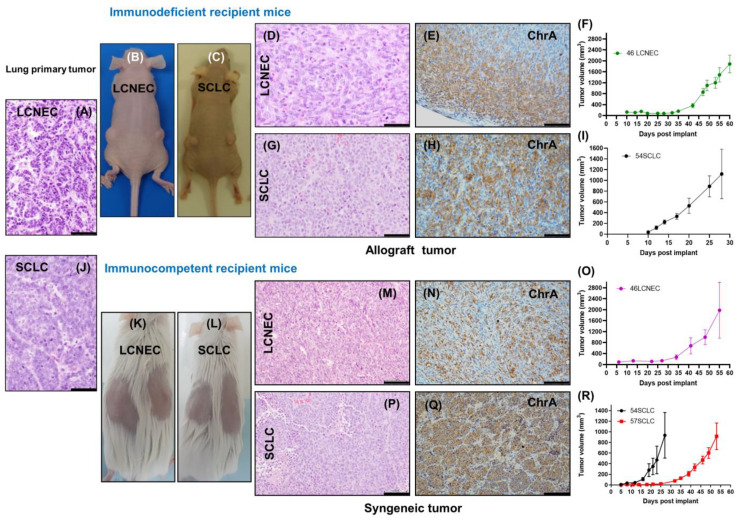
Tumorigenicity of the LCNEC and SCLC cell lines. H&E staining of the lung primary LCNEC (**A**) and SCLC (**J**) spontaneous primary tumors. Representative macroscopic images of LCNEC (**B**,**K**) and SCLC (**C**,**L**) subcutaneous tumors growing in NMRI nude (**B**,**C**) or QKO (**K**,**L**) mice. Representative photomicrographs of H&E staining (**D**,**G**,**M**,**P**) of LCNEC (**D**,**M**) and SCLC (**G**,**P**) and IHC staining (**E**,**H**,**N**,**Q**) of the neuroendocrine marker chromogranin A in LCNEC (**E**,**N**) or SCLC (**H**,**Q**) arising in immunodeficient (**D**,**E**,**G**,**H**) or immunocompetent (**M**,**N**,**P**,**Q**) mice. Subcutaneous transplant tumor growth curves (**F**,**I**,**O**,**R**). Average tumor volume of subcutaneous tumors from 46LCNEC in NMRI nude mice (*n* = 5, passage 17) (**F**); 54SCLC in NMRI nude mice (*n* = 5, passage 22) (**I**); 46LCNEC in syngeneic mice (*n* = 6, passage 17) (**O**); 54SCLC and 57SCLC in syngeneic mice (*n* = 6, passage 22 and *n* = 15, passage 16, respectively) (**R**). Error bars correspond to SEM. Scale bars = 50 µm.

Cell lines were injected subcutaneously into mice to test their tumorigenicity. We first evaluated the 46LCNEC and 54SCLC cell lines in an immunodeficient background. Cells were transplanted into both flanks of NMRI nude mice and they generated allograft tumors (Figure 2A–I). Both LCNEC and SCLC cells showed strong growth capacity in vivo, particularly the 54SCLC cells with a tumor volume reaching about 1100 mm^3^ at day 28 post-injection (Figure 2I). Subsequently, syngeneic grafts were developed in the QKO mice: the 46LCNEC, 54SCLC, and 57SCLC cell lines were injected under the dermis-generated tumors in the recipient mice at different rates depending on the cell line (Figure 2O,R) beginning at day 8 (54SCLC) after injection and reaching a volume of about 900 mm^3^ after 27 days. We compared the tumorigenic potential of LCNEC and SCLC cell lines in either immunodeficient (MMRI nude) or immunocompetent (QKO) backgrounds and observed that both the 46LCNEC and 54SCLC subcutaneous tumors developed from the same cell passage reached a similar size irrespective of the recipient mice (Figure S4).

**Figure 3:**
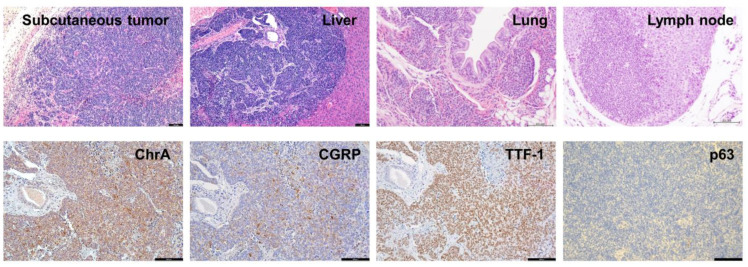
Metastatic ability of SCLC cell lines in syngeneic mice. The upper panel shows representative H&E staining of a 57SCLC syngeneic tumor and metastasis in liver, lung, and lymph node as quoted (scale bars = 50 µm (images on the left), 200 µm (images on the right)). In the lower panel, immunohistochemistry staining for the quoted proteins is depicted in a liver metastasis of 57SCLC (scale bars = 100 µm).

The morphology of the induced tumors is shown in Figure 2. Cells derived from SCLC tumors growing in suspension generated tumors composed of small cells, scarce cytoplasm, and SCLC characteristics (Figure 2G,P), while adherent cells derived from LCNEC tumors generated tumors with a large cell phenotype and visible cytoplasm (Figure 2D,M). Thus, allograft and primary tumors showed similar morphologies and neuroendocrine marker profiles (Figure 2A,D–H,J,M–Q). Vascularization was present in SCLC subcutaneous tumors (Figure 2G,P and Figure 3). Subcutaneous tumors reproduced the characteristic neuroendocrine antigen profile of the primary tumors from which they were derived and were coincident with high-grade neuroendocrine lung carcinomas. They showed positive staining of neuroendocrine markers such as Chr A and CGRP or the thyroid transcription factor TTF-1 and were negative for CC-10 (Clara or club cell secretory protein) and p63 (squamous cell carcinoma) (Figure 2E,H,N,Q and Figure S5). 

As shown in Figure S2 and as was observed in the cell lines, tumors retained the mutant alleles in the genes *Rb1*, *Rbl1*, *Pten*, and *Trp53* of the primary tumors from which they were initially derived. 

### 2.3. The SCLC Syngeneic Tumors Showed the Ability to Form Metastasis

Given the high metastatic rates reported in human SCLC patients (about 70% at diagnosis) [23], we sought to assess the metastatic ability of 54SCLC and 57SCLC allograft syngeneic tumors. We had previously observed in the K5QKO model, which developed spontaneous SCLC tumors upon deletion of the four tumor suppressor genes *Rb1*, *Rbl1*, *Pten*, and *Trp53* [20,21], the appearance of metastases in the liver and lymph nodes [21]. Thus, we analyzed the metastatic potential of the cell line models and the subcutaneous tumors arisen from them. Histologic analysis and staining with neuroendocrine markers confirmed that syngeneic mice injected with the 57SCLC cell line developed metastases in the lungs, liver, and lymph nodes (Figure 3) (*n* = 5 out of 22 mice) and showed the ability of these cell-derived tumors to colonize distant organs, thus mimicking the primary tumors arisen in the lungs and the human condition. As tumors injected with 54SCLC grew very rapidly in the syngeneic mouse (see Figure 2 and Figure S4), we surgically removed the subcutaneous tumor when it reached a size >1400 mm^3^ and allowed its regrowth. Mice were sacrificed when regrown tumors again reached a size >1400 mm^3^, and we observed the colonization of distant organs such as the lungs and lymph nodes (*n* = 3 out of 3). Immunohistochemical detection of the neuroendocrine markers chromogranin A or CGRP as well as the expression of TTF-1 or the absence of p63 that were observed in subcutaneous tumors (Figure 2 and Figure S5) corroborated the presence of metastatic tumor cells (Figure 3).

These data show the ability to form metastases of both the 54 and 57SCLC cell lines in the syngeneic model, which reproduces the pattern observed in human SCLC and makes these cell lines particularly suitable to address anti-metastatic therapeutic approaches in defined molecular settings.

### 2.4. Transcriptional Profile of Mouse LCNEC and SCLC Cell Lines and Allografts

**Figure 4:**
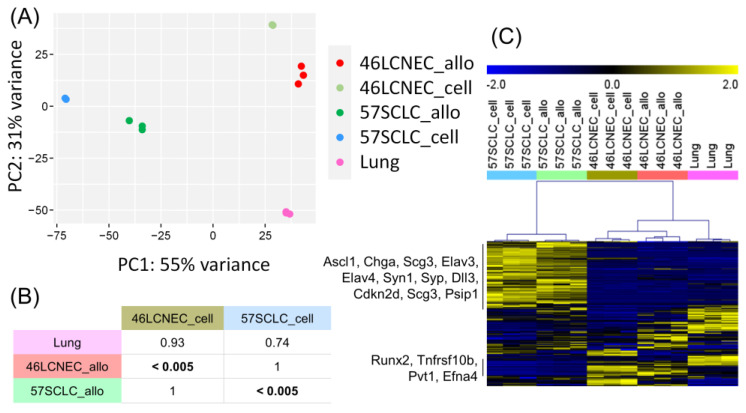
Genomic characterization of mouse LCNEC and SCLC cell lines and allografts. (**A**) PCA analysis showed that triplicates from each sample type grouped together in the bi-dimensional space. (**B**) Subclass association table for cell lines, allograft tumors, and lung samples. 46LCNEC_cell: 46LCNEC cell line; 57SCLC_cell: 57SCLC cell line; 46LCNEC_allo: 46LCNEC syngeneic allograft tumor; 57SCLC_allo: 57SCLC syngeneic allograft tumor; Lung: normal lung tissue from littermates. Fisher’s statistics, FDR-corrected *p*-values for the indicated combinations. (**C**) Heatmap representing the expression values of the LCNEC/SCLC classifier. Yellow and blue indicate high and low expression, respectively. Unsupervised hierarchical clustering, Spearman rank correlation, complete linkage. Selected candidate genes typically enriched in human SCLC or LCNEC are shown on the left.

To further characterize our models, we performed RNA sequencing from LCNEC and SCLC cell lines (46LCNEC_cell, 57SCLC_cell), their corresponding derived syngeneic allograft tumors (46LCNEC_allo, 57SCLC_allo), and normal lung tissue from littermates used for 57SCLC and 46LCNEC syngeneic allografts (lung). As expected, principal component analysis (PCA) showed that triplicates from each sample type grouped together in the bi-dimensional space (Figure 4A). To analyze the degree of similarity between the cell lines and their allograft tumors, we used subclass mapping (SubMap) from GenePattern [24,25]. SubMap is an unsupervised method that bidirectionally compares subclasses by evaluating the significance of the association. This method identified that gene expression from cells and allograft tumors of the same subtype displayed a similar transcriptome program (Figure 4B). Next, we used a gene classifier (Supplementary Dataset S1) capable of discriminating human LCNEC-SCLC subtypes [6] to perform unsupervised clustering of our cell and allograft tumor models (Figure 4C). This classifier clustered together cell and allograft tumor samples from each subtype and was able to separate LCNEC and SCLC models. Neuroendocrine genes typically expressed at a high level in SCLC such as Ascl1, Dll3, Syp, Elav3, Elav4, and Chga were specifically up-regulated in SCLC cells and allograft tumors. Conversely, immune response (Tnfrsf10b) and transcription factor (Runx2) genes found in human LCNEC [6] exhibited high levels of expression in LCNEC cells and allograft tumors.

**Figure 5:**
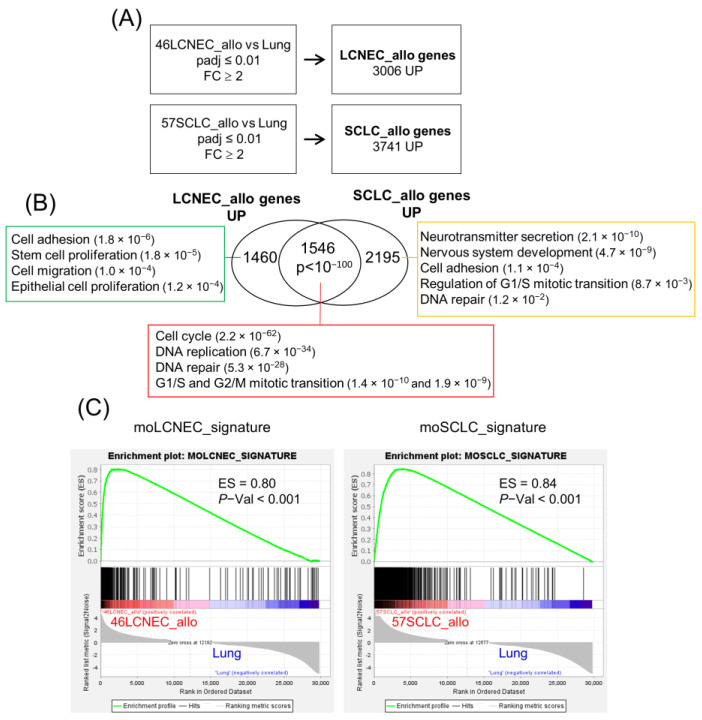
Biological pathways enriched in mouse LCNEC and SCLC syngeneic allografts. (**A**) LCNEC_allo and SCLC_allo genes: genes significantly (padj Val ≤ 0.01) up-regulated more than 2-fold in 46LCNEC_allo or 57SCLC_allo samples compared to Lung (non-tumor lung tissue). Numbers indicate gene symbol identifiers. Padj: adjusted *p*-value, Benjamini–Hochberg method; FC: fold change. (**B**) Gene ontology analysis of 46LCNEC and 57SCLC allograft syngeneic tumors. Venn diagram shows the overlap between LCNEC_allo and SCLC_allo genes. Hypergeometric test was used to assess the statistical significance of the overlap. The rectangular boxes contain the main signaling pathways enriched in the indicated groups (Gene Ontology Biological Processes (GOBP) Direct). The *p* values in brackets. (**C**) Gene set enrichment analysis (GSEA) plots showing enrichment in the LCNEC and SCLC allograft tumors in moLCNEC and moSCLC gene signatures established in the corresponding CMV-QKO and K5-QKO mouse models. Note that the positions of the moLCNEC and moSCLC genes are significantly skewed toward the left end of the rank-sorted list, reflecting their statistically significant induction in LCNEC and SCLC allograft tumors. Size: number of genes in the gene set after filtering out those genes not in the expression dataset. Positive enrichment score (ES: from 0 to 1) reflects the degree to which a gene set is overrepresented at the top of a ranked list of genes in the expression dataset. The *p*-value is the probability of obtaining an enrichment score from the actual ranking bigger than that for the random permutations.

We evaluated whether our cell and allograft syngeneic models resembled the spontaneous tumors from which they originated. In a previous work [21], we characterized the genomic profile of SCLC and LCNEC spontaneous tumors in the CMV-QKO and K5-QKO mouse models used to generate the cell lines and the syngeneic allograft tumors described here. We analyzed the biological pathways enriched in CMV-QKO LCNEC and K5-QKO SCLC tumors and established gene signatures of each tumor type (moLCNEC_signature and moSCLC_signature, respectively) (Supplementary Dataset S2). Here, we compared 46LCNEC_allo and 57SCLC_allo tumor samples with non-tumor lung tissue (Lung) to find genes up-regulated in these tumors (Figure 5A) and analyzed the biological pathways shared or specific for each tumor type. Similar to the CMV-QKO and K5-QKO spontaneous tumors, up-regulated genes common to both allograft tumors are significantly involved in cell cycle and mitotic progression. Like the K5-QKO SCLC tumors from which they originated, neuroendocrine markers and nervous system pathways were significantly present in 57SCLC_allo tumors (Ascl1, Chga, Scg3, Syp, Elav3, Elav4). The 46LCNEC_allo tumors also showed similarity with their parental CMV-QKO tumors (Figure 5B; Supplementary Dataset S3). Moreover, gene set enrichment analysis (GSEA) revealed significant enrichment in the CMV-QKO moLCNEC_signature and K5-QKO moSCLC_signature in their corresponding allograft tumors (Figure 5C) and cell lines (Figure S6).

## 3. Discussion

This study presents new mouse LCNEC and SCLC cell lines, which were generated from spontaneous tumors arisen in our previously generated GEMMs [20,21]. Subcutaneous injection of these cells resulted in allograft tumors and the development of syngeneic models for further investigation of high-grade neuroendocrine lung cancer that, in the case of SCLC, also represents a tool to study metastatic processes. We described here the first murine cell line model of LCNEC. Moreover, because of the lack of high-grade neuroendocrine lung carcinoma material from surgery, laboratory research studies are limited, which is more significant in the case of LCNEC. In contrast, cell lines provide a limitless source of materials that could be shared by laboratories worldwide and provide basic and translational resources for the study of high-grade neuroendocrine lung cancer pathogenesis. In fact, cancer cell lines remain the most widely used models in cancer biology research [26] and are the primary resource for studying SCLC [10].

Genetically engineered mouse models (GEMMs) faithfully reproduce human tumors in a physiological and immunocompetent environment [14]. However, many GEMMs develop multifocal tumors of different stages of progression (including low-grade malignancy) [13,27] that may bias any assessments of antitumoral responses. The high-grade neuroendocrine cancer cell lines developed here reproduced the aggressive primary tumors from which they originated. As these cells lines can develop allograft tumors in both immunodeficient and immunocompetent mouse environments, they constitute a suitable tool for preclinical analysis in both backgrounds. These tumors offer other advantages such as growth at short latency times with almost full penetrance, control of the number of tumors per mouse, easy monitoring of growth and therapeutic responses, and reduced costs. The development of syngeneic LCNEC and SCLC models featuring murine immunity and stroma offers the possibility of therapeutic assessment of new immunotherapies, either alone or in combination strategies, which would provide relevant information needed for future clinical trials. 

The relevance of mouse cell lines for basic, translational, and preclinical research depends on how closely they resemble their human tumors’ counterparts. The comparative transcriptomic analyses performed here shows that LCNEC and SCLC cells and subcutaneous tumors retain the transcriptomic profile of the original spontaneous tumors and share strong similarities with those of human LCNEC and SCLC [6,21,28]. They therefore provide a suitable framework to test anti-cancer therapies in vitro and in vivo. 

SCLC is a highly metastatic and recalcitrant carcinoma, with metastasis as a major cause of mortality. While human SCLC cells fail to metastasize when transplanted into mice, few murine SCLC cell lines have been reported to generate metastasis after subcutaneous engraftment [23,29] to serve the purpose of dissecting molecular mechanisms of metastatic development such as cell–cell interactions between NE and non-NE cells in SCLC cells [30,31] or prove the critical role of Nfib in promoting metastasis in SCLC [32]. The SCLC cell lines presented here displayed the ability to spontaneously colonize distant organs from the subcutaneous tumor and to form metastasis with no need of inoculating cells via alternate routes (such as intravenous, intracranial, intraperitoneal, or orthotropic sites). These features make them valuable tools to identify molecular and cellular mechanisms of SCLC metastasis and to assess potential agents against metastatic programs.

In summary, we report the establishment and precise characterization of high-grade neuroendocrine lung carcinoma (LCNEC and SCLC) cell lines and transplanted tumors in different backgrounds. To our knowledge, these are the first murine LCNEC cell lines that have been generated. Our data support the neuroendocrine properties of these novel cell lines and their transcriptomic similarities among cells, allograft, and spontaneous and human tumors of the same cancer type (LCNEC and SCLC). Importantly, the SCLC cell lines showed metastatic potential with the ability to grow metastatic lesions. In summary, we showed that these cell lines might serve as reliable model systems for further neuroendocrine lung cancer research and identification of novel molecular targets and immunotherapies for the treatment against highly aggressive SCLC and LCNEC.

## 4. Materials and Methods

### 4.1. Donor and Recipient Mice 

All the animal work was approved by the Animal Ethical Committee (CEEA) and conducted in compliance with Centro de Investigaciones Energéticas, Medioambientales y Tecnológicas (CIEMAT) guidelines.

QKO mice bear floxed *Rbl1*, *Pten*, and *Trp53* alleles along with a null *Rbl1* gene and have been described elsewhere [21]. Primary tumors were generated after adenovirus Ad-CMVcre (CMV-QKO) or Ad-K5cre (K5-QKO) intratracheal infection. NMRI-Foxn1^nu/nu^ immunodeficient 6–8-week-old mice were obtained from Janvier (Saint-Berthevin). 

### 4.2. Establishment of SCLC and LCNEC Murine Cell Lines and Cell Culture 

For the isolation and culture of tumor cells, CMV-QKO and K5-QKO mice [21] were sacrificed, their airway systems were removed, and primary tumors were dissected from lung tissues and minced with a razor blade. Cells were disaggregated by treatment with collagenase 0.25% for 1.5 h at 37 °C and mechanical ablation with a plastic pipette and passed through a 100 μm sieve. Then, cells were washed twice with PBS and seeded in RPMI medium (Gibco, Thermo Fisher Scientific, Waltham, MA, USA) supplemented with 10% FCS, 1% antibiotic-antimycotic 100× (Gibco), 1% ITS (Gibco), 0.005% mEGF (MilliporeSigma Merck, Burlington, MA, USA), and 0.4% hydrocortisone (MilliporeSigma).

Cells grew either in suspension (SCLC cells from K5-QKO tumors) or in adherent monolayer (LCNEC cells from CMV-QKO tumors). To split the cell cultures, suspending aggregates were harvested and mechanically disaggregated by pipetting with micropipette. Adherent cultures were washed (PBS) and detached by incubating the cells in trypsin-EDTA solution (MilliporeSigma) for 3 min at 37 °C.

To assess cell growth kinetics, 100,000 LCNEC or SCLC cells were seeded into 75 cm^2^ flasks and cultured for 5 days. Every day, the cell number was counted in a Countess 3 Automated Cell Counter (Invitrogen, Thermo Fisher Scientific, Waltham, MA, USA). 

All experiments were performed with mycoplasma-free cells.

### 4.3. Evaluation of Tumorigenic and Metastatic Potential 

To assay tumor formation ability, 200,000 LCNEC and SCLC cells were suspended in 100 μL of a 1:1 mixture of medium and Matrigel (Corning, Merck, Burlington, MA, USA) and injected subcutaneously into the flanks of recipient (immunodeficient NMRI nude or immunocompetent QKO) mice. Tumors were measured twice a week with a caliper, and tumor volume was calculated with the formula (4 π ((long side/2)^2^) × (short side/2))/3. Tumors were allowed to grow until a maximum volume of 1500 mm^3^; then, mice were euthanized and processed for histology. Complete necropsies were performed in search of metastases. For mice injected with 54SCLC, subcutaneous tumors were surgically excised and allowed to regrow. Organs were processed for histology, as described below (see Section 4.6).

### 4.4. Genotyping 

Genomic DNA was isolated from *Rb1^F/F^*, *Rbl1^-/-^*, *Trp53^F/F^*, and *Pten^F/F^* control lungs, cells, and tumors using DNeasy Blood & Tissue Kit (Qiagen, Venlo, The Netherlands). Primer sequences, amplified fragments, and PCR amplification product sizes appear in Table S1. 

### 4.5. Immunofluorescence 

Adherent cell lines (LCNEC) were seeded in chamber slides and grew there for 48 h. Floating cell lines (SCLC) were spun in Shandon CytoSpin 2 centrifuge at 400 rpm for 5 min. For the immunofluorescence analyses, the cells were (1) fixed with MetOH-acetone (1:1) at −20 °C for 10 min; (2) incubated with horse serum 10% for 1 h at room temperature; and (3) washed three times with sterile phosphate-buffered saline (PBS) (pH 7.5) prior to incubation with the appropriate primary antibodies diluted in horse serum 10%. Primary antibodies were used as follows: 1/200 dilution of anti-NCAM CD56 (AB5032, MilliporeSigma); 1/100 dilution of anti-SSTR2 (HPA007264, MilliporeSigma); and 1/2000 dilution of anti-CGRP (C8198, MilliporeSigma). Secondary antibodies were used as follows: 1/1000 anti-rabbit AlexaFluor488 (A11008, Molecular Probes. Thermo Fisher Scientific, Waltham, MA, USA) and 1/1000 anti-mouse AlexaFluor488 (A11001Molecular Probes). Diamidinophenylindole (DAPI) was used to counterstain the nuclei or chromosomes.

### 4.6. Histology and Immunohistochemistry 

Necropsies were performed at the end of the experiments. Upon necropsy, tumors and tissues were fixed in formalin for 24 h. Fixed tissues were dehydrated and embedded in paraffin wax. Sections (5 μm) were stained with hematoxylin and eosin (H&E) for histological analysis or processed for immunohistochemistry. Immunohistochemical analyses were performed as follows: the sections were (1) deparaffinized; (2) incubated with 10% horse serum for 30 min at 37 °C to block non-specific binding; and (3) washed three times with sterile phosphate-buffered saline (PBS) (pH 7.5) prior to incubation with the appropriate primary antibodies diluted in horse serum 10%. Primary antibodies were used as follows: 1/100 dilution of anti-chromogranin A (ab15160, Abcam, Cambridge, UK); 1/2000 dilution of anti-CGRP (C8198, MilliporeSigma); 1/200 dilution of anti-TTF1 (ab76013, Abcam, Cambridge, UK); 1/50 dilution of anti-p63 (ab53039-100, Abcam, Cambridge, UK); and 1/100 dilution of anti-CC10 (sc-25554, Santa Cruz Cruz Biotecnology, Dallas, TX, USA). Secondary antibodies were used as follows: 1/1000 biotin anti-mouse (No. 715-065-151, Jackson ImmunoResearch, West Grove, PA, USA) and 1/1000 biotin anti-rabbit (No. 711-065-152, Jackson ImmunoResearch).

### 4.7. RNA Isolation and Gene Expression Profiling 

Cells and tissues were embedded in RNALater (Ambion Inc., Thermo Fisher Scientific, Waltham, MA, USA, and RNA was isolated and purified using miRNeasy Mini Kit (Qiagen) according to the manufacturer’s instructions. RNA yield and quality were determined using an Agilent 2100 Bioanalyzer.

Transcriptome sequencing was performed from *n* = 3 57SCLC cell line samples, passage 11, from 3 different cell flasks; *n* = 3 46LCNEC cell line samples, passage 17, from 3 different cell dishes; *n* = 3 57SCLC syngeneic allograft tumors (from transplanted cell passage 11); *n* = 3 46LCNEC syngeneic allograft tumors (from transplanted cell passage 17); and *n* = 3 normal lung tissue from littermates of mice used for 57SCLC and 46LCNEC syngeneic allografts. RNA sequencing was performed after poly-A selection using Illumina NovaSeq 2 × 150 bp sequencing. Reads were aligned to GRCm39 mouse genome with “Rbowtie2”, counts extracted with featureCounts function from “Rsubread” and gencode annotation release 27 (gencode.vM27.annotation.gtf), and differential expression with DESeq2. Principal component analysis graph was plotted after variance stabilizing transformation (vst function in DESeq2). 

### 4.8. Gene Set Enrichment Analysis 

We developed signatures of genes specifically up-regulated in tumors from our CMV-QKO LCNEC (moLCNEC) and K5-QKO SCLC (moSCLC) models [21]. Briefly, Affymetrix probe set identifiers for genes significantly up-regulated (FDR ≤ 0.01; ≥2-fold) in moLCNEC versus normal lung (434 Affy IDs) or moSCLC versus normal lung (1335 Affy IDs), and these were manually curated to generate two gene signatures: moLCNEC_signature and moSCLC_signature (Supplementary Dataset S2). Gene set enrichment analysis (GSEA, www.broadinstitute.org/gsea (accessed on 5 May 2023)) [33] was used to analyze enrichment of these gene signatures in the LCNEC and SCLC allograft tumors. GSEA was performed from normalized counts after removal of genes with counts = 0 in all samples.

### 4.9. Subclass Association 

We analyzed the association between our cells and allograft RNAseq expression data using the SubMap module of Gene Pattern [24,25]. SubMap uses GSEA to measure the similarity between the gene expression profiles of subclasses. The output of this analysis is a subclass association (SA) table with the *p*-values for each SA. The SubMap module uses permutations to compute *p*-values. Number of permutations for nominal-p of Fisher’s statistics: 1000. The *p*-value correction method: FDR and Bonferroni.

### 4.10. Human LCNEC/SCLC Classifier 

The 300-gene LCNEC/SCLC classifier from George et al. (2018) [6] was converted into a 275-gene mouse classifier (Supplementary Dataset S2) using DAVID Gene ID Conversion Tool. Next, we used MeV v4.9 for the unsupervised hierarchical clustering analysis of the samples (Spearman rank correlation, complete linkage).

### 4.11. Analysis of Biological Pathways 

DESeq2 [34] was used to identify genes that were differentially expressed in the 46LCNEC_allo and 57SCLC_allo tumors versus non-tumor lung (Lung). Genes having an adjusted *p*-value threshold of ≤0.01 and an expression fold change (FC) ≥2 were selected. DAVID [35] web server (https://david.ncifcrf.gov/home.jsp (accessed on 5 May 2023)) from the NIAID/NIH was used to identify Gene Ontology Biological Process (GOBP) functional categories (GOTERM_BP_DIRECT). GOBP terms were listed by *p*-value based on EASE score [36] and manually curated (Supplementary Dataset S3).

### 4.12. Data and Statistical Analyses 

Data were analyzed with GraphPad Prism 9 software (GraphPad Software, La Jolla, CA, USA). Statistical analysis was performed using U Mann–Whitney test.

## 5. Conclusions

Patients with SCLC or LCNEC have very poor prognoses, low survival rates, and few effective treatments. Surgery is not usually an option and, as a consequence, human material for research is scarce. In an attempt to address these issues, this study described newly generated mouse cell lines and in vivo models of SCLC and LCNEC that closely resemble their human counterparts. These constitute relevant tools for further basic and translational research as well as preclinical testing of potential antitumor compounds, including immunotherapies. It is of note that no mouse LCNEC cell lines have been previously described.

## Data Availability

All the transcriptome-sequencing data are available at Gene Expression Omnibus ((GEO) http://www.ncbi.nlm.nih.gov/geo/, accession number GSE242422 (access release date 6 September 2024)).

## Supplementary Materials

The following supporting information can be downloaded at: https://www.mdpi.com/article/10.3390/ijms242015284/s1.
