# Supplementary material for: Novel Mouse Cell Lines and In Vivo Models for Human High-Grade Neuroendocrine Lung Carcinoma, Small Cell Lung Carcinoma (SCLC), and Large Cell Neuroendocrine Carcinoma (LCNEC)
Source: Int J Mol Sci. 2023 Oct 18;24(20):15284. doi: 10.3390/ijms242015284 (PMC10607103; doi:10.3390/ijms242015284)
Supplement: Supplementary file 1 [file ijms-24-15284-s001.zip › Recuero et al Supplementary Table S1.pdf]

Table S1. PCR primer sequences for genotyping

Primer Name	Primer Sequence (5`-3`)	Product Size (bp)
**p53 10 F**	AAGGGGTATGAGGGACAAGG	Wt 154 bp (10F-10R2)
**p53 10R2**	GAGACAGGGTCTTGCTATTGT	Floxed and 102 (loxP F-10R2) 307 (10F-10R2)* bp
**p53 loxP F**	CGAAGTTATTAGGTCCCTCGAA	
**p53 1F2**	AGAAAGGGCGACTGACTGTG	
**Rb 18**	GGCGTGTGCCATCAATG	Wt 680 bp
**Rb 19**	AACTCAAGGGAGACCTG	Floxed 748 bp
		Deleted 300 bp
**PTEN loxP F**	AGTGGCATGTTTTGTCTATGGT	Wt 72 bp
**PTEN loxP R**	ACGAGTCCTCTGAAAAAGCAGT	Floxed 118 bp
**PTEN Del**	TGGGGCTGCAGGAATTCGATA	Deleted 141 bp (Del-loxP R)
**p107 F2**	GCAACTTTGGTGGCTCTTCAT	Wt 130 bp
**p107 R2**	CCACAAGAGTTTCGTGAGCG	
**p107 LzR**	GGGCTGCAGGAATTCGATA	Deleted 78 bp
