# Supplementary figures and images for: Novel Mouse Cell Lines and In Vivo Models for Human High-Grade Neuroendocrine Lung Carcinoma, Small Cell Lung Carcinoma (SCLC), and Large Cell Neuroendocrine Carcinoma (LCNEC)
Source: Int J Mol Sci. 2023 Oct 18;24(20):15284. doi: 10.3390/ijms242015284 (PMC10607103; doi:10.3390/ijms242015284)

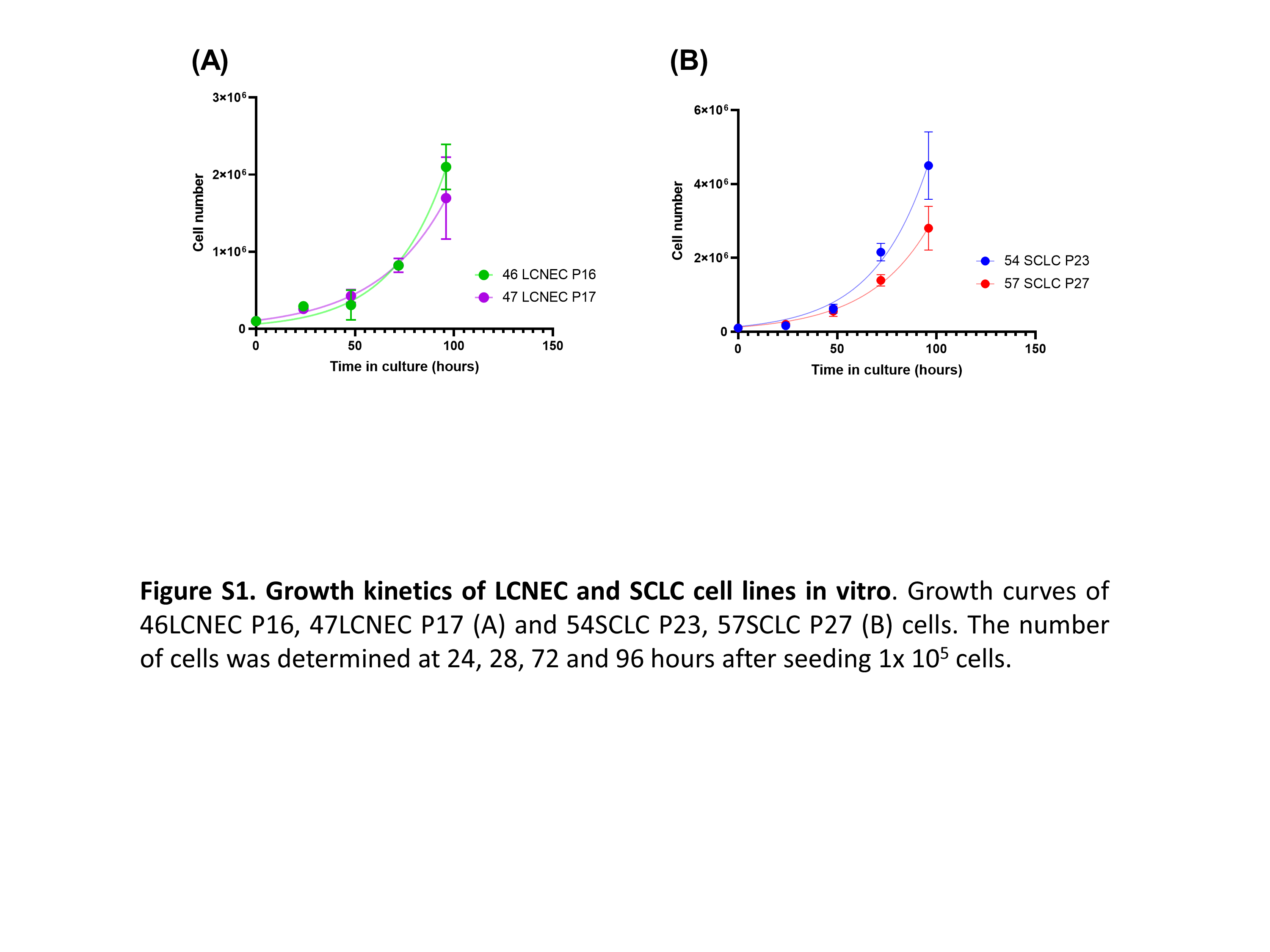

Supplement: Supplementary file 1 [file ijms-24-15284-s001.zip › Recuero et al Supplementary Figure S1.TIF]

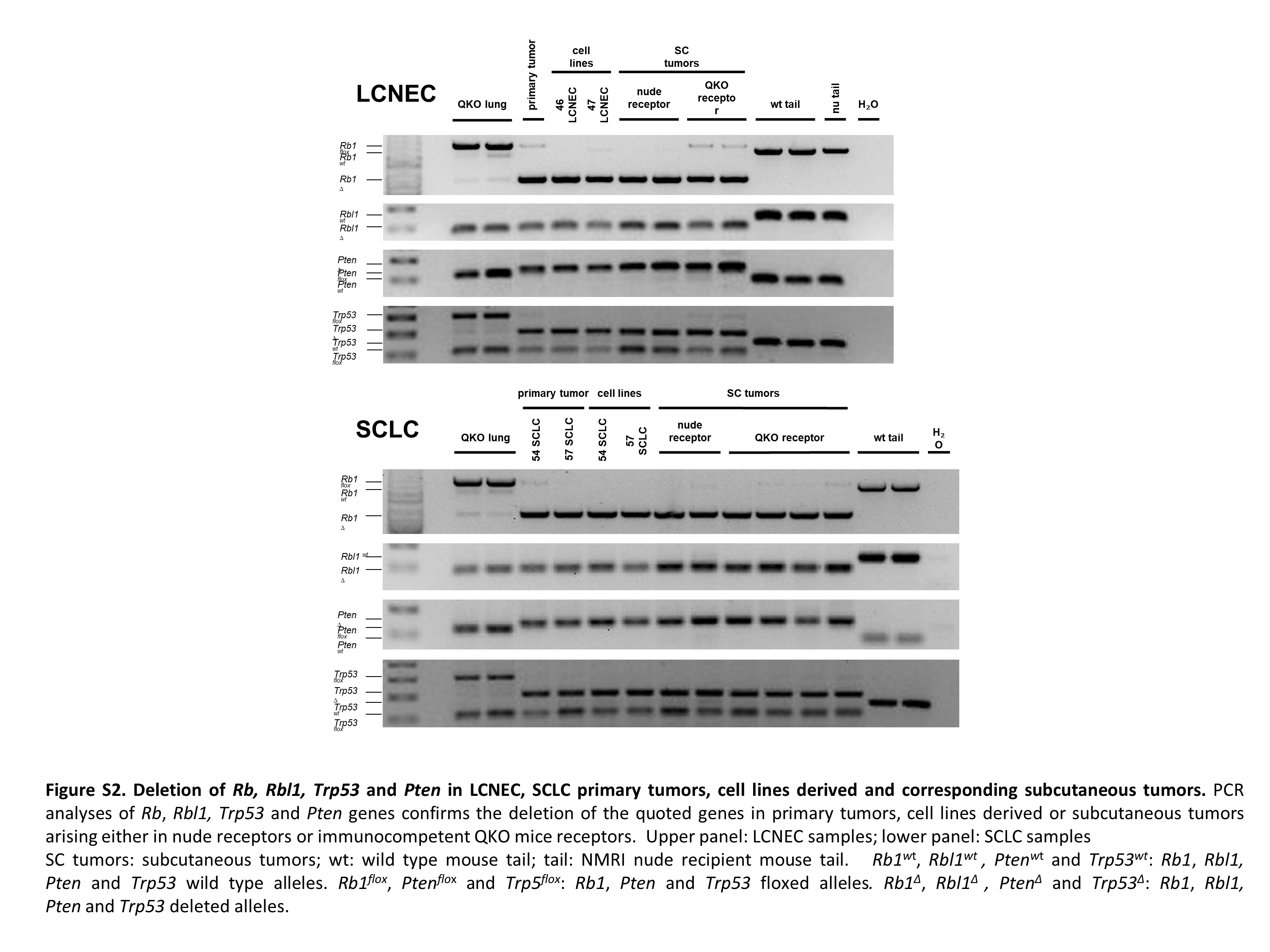

Supplement: Supplementary file 1 [file ijms-24-15284-s001.zip › Recuero et al Supplementary Figure S2.TIF]

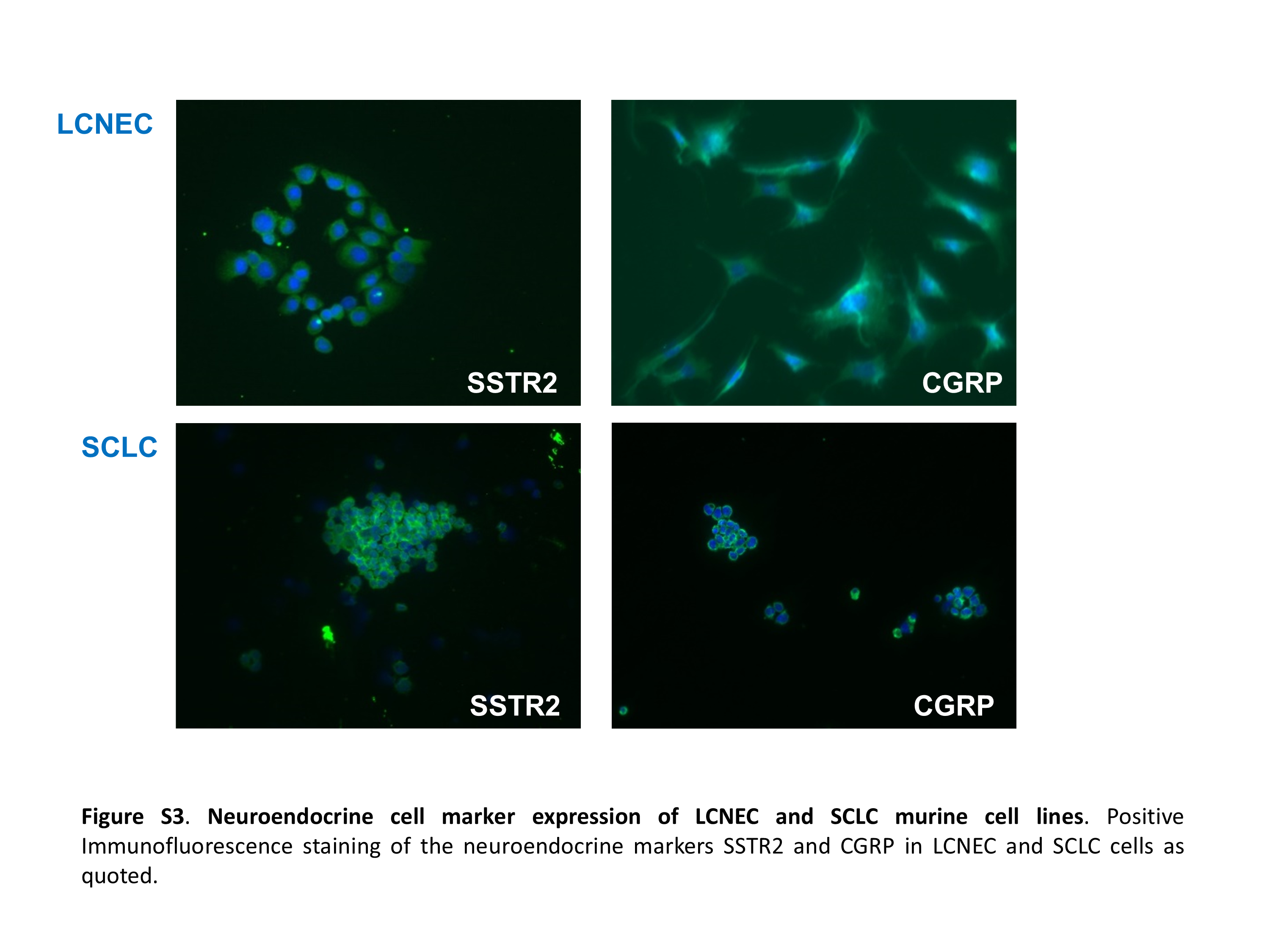

Supplement: Supplementary file 1 [file ijms-24-15284-s001.zip › Recuero et al Supplementary Figure S3.TIF]

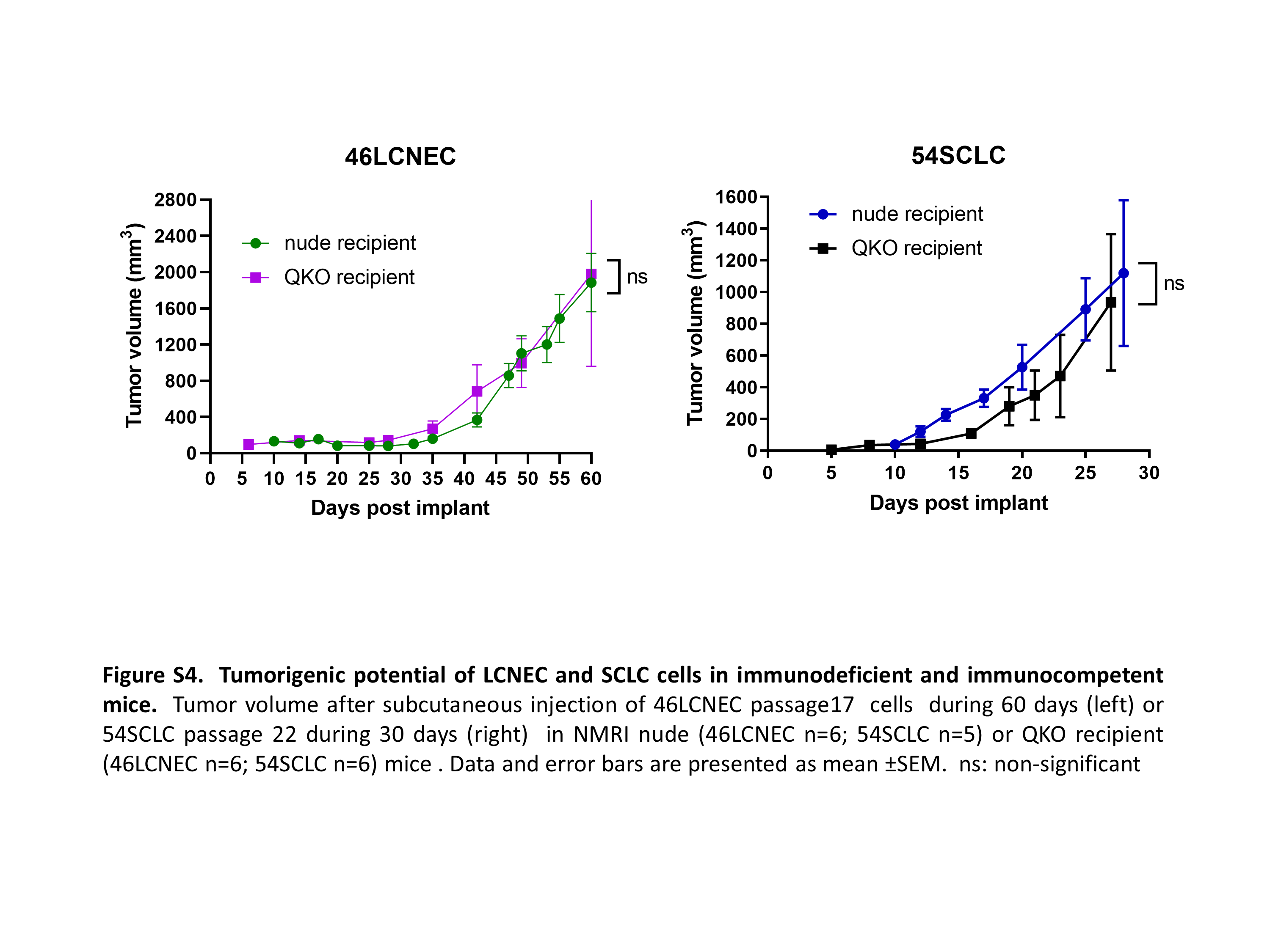

Supplement: Supplementary file 1 [file ijms-24-15284-s001.zip › Recuero et al Supplementary Figure S4.TIF]

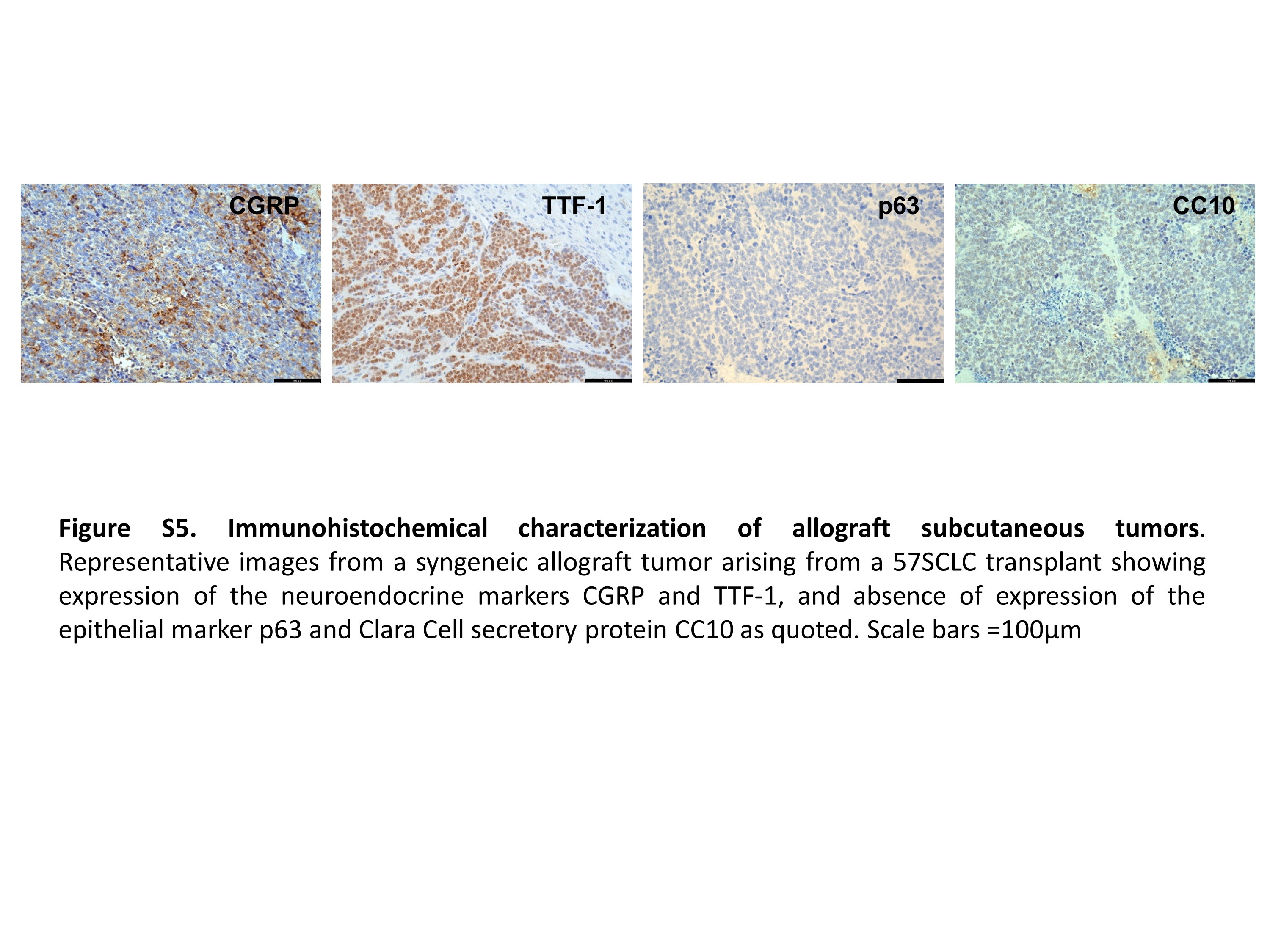

Supplement: Supplementary file 1 [file ijms-24-15284-s001.zip › Recuero et al Supplementary Figure S5.TIF]

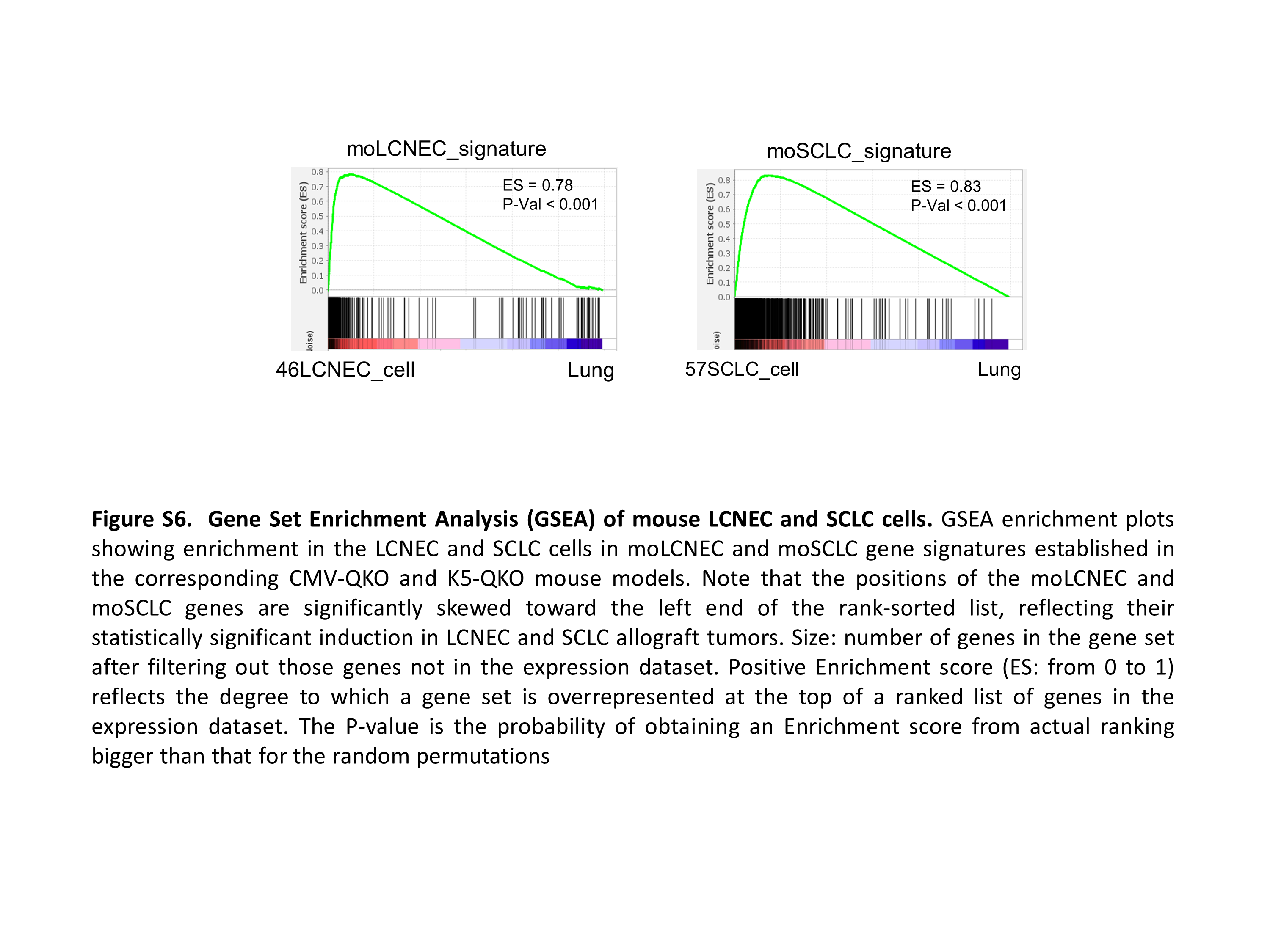

Supplement: Supplementary file 1 [file ijms-24-15284-s001.zip › Recuero et al Supplementary Figure S6.TIF]
